# Two rare schwannomas of head and neck

**DOI:** 10.1186/1746-1596-9-27

**Published:** 2014-02-05

**Authors:** Karima Idrissi Serhrouchni, Laila Chbani, Nawal Hammas, Dounia Kamal, Hinde El Fatemi, Taoufik Harmouch, Noure-eddine El Alami, Afaf Amarti

**Affiliations:** 1 Department of Pathology, Hassan II University Hospital of Fez, Hassan, Morocco; 2 Department of Otology and Laryngology, Hassan II University Hospital of Fez, Hassan, Morocco

**Keywords:** Schwannoma, Facial nerve, Nasal cavity, Parotid gland

## Abstract

Twenty-five to 45 percent of all schwannomas occur in the head and neck. Most of them arise along the vestibular portion of the eighth cranial nerve (acoustic neurinoma). They rarely originate from the peripheral facial nerve or other nerves within the parotid gland. Less than 4% of schwannomas involve the nasal cavity and paranasal sinuses. They arise from the branches of the trigeminal nerve and autonomic nervous system.

We report two cases of schwannomas arisng from intraparotid facial nerve and nasal cavity. The first case diagnosed in a 62-year-old-man presented with 2-year-history of painless mass of the parotid gland. The lesion was found to be cystic through the pre operative examinations and investigations. The histology of the specimen retained an intraparotid cystic schwannoma. The second case concerned a 75-year-old-man presented with episodes of nasal obstruction, rhinorrhea, anosmia and headache. Histological study of the specimen showed fusiform cells with strongly and diffusely immunostaining for S100 protein suggesting a schwannoma.

**Virtual Slides:**

http://www.diagnosticpathology.diagnomx.eu/vs/1098335216112242.

## Background

Schwannomas of the facial nerve can occur at any point along its complicated anatomical course from the cerebellopontine angle to its multiple peripheral branches in the face. They originate rarely from the peripheral facial nerve or other nerves within the parotid gland and they are typically present as an asymptomatic parotid mass [1]. Schwannoma involves the nasal cavity and paranasal sinuses represent less than 4% and they occur in middleaged adults with an equal gender distribution [2]. The cases are of interest due to the relative rarity of the pathology and presence of non-significant symptoms for a presumed initial clinical diagnosis.

## Case reports

### Case 1

**Figure 1:**
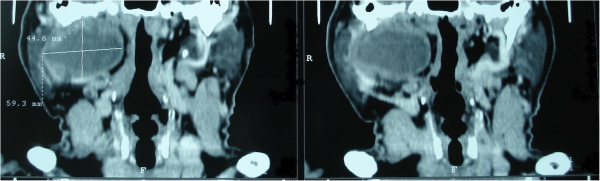
Computed tomography scan (coronal cuts): Homogenous and well defined cystic mass involving of the right parotid gland and measuring 44.6 mm × 59.3 mm.

A 62-year-old man, presented with 2 years history of a painless and gradually enlarging right parotid cystic mass. He denied any facial weakness, twitching or pain. There was no history of prior irradiation or trauma or smoking. Examination revealed a 5 × 4 cm cystic non-tender mobile mass. Facial nerve function as well as the remainder of head and neck examination was normal. The neck ultrasonography showed a hypoechoic and homogeneous right intraparotidien process, though limited, measuring 6 cm of diameter. Dental panoramic showed osteolysis of the right mandible and computed tomography confirmed the origin of the intra-parotid lesion (Figure 1).

**Figure 2:**
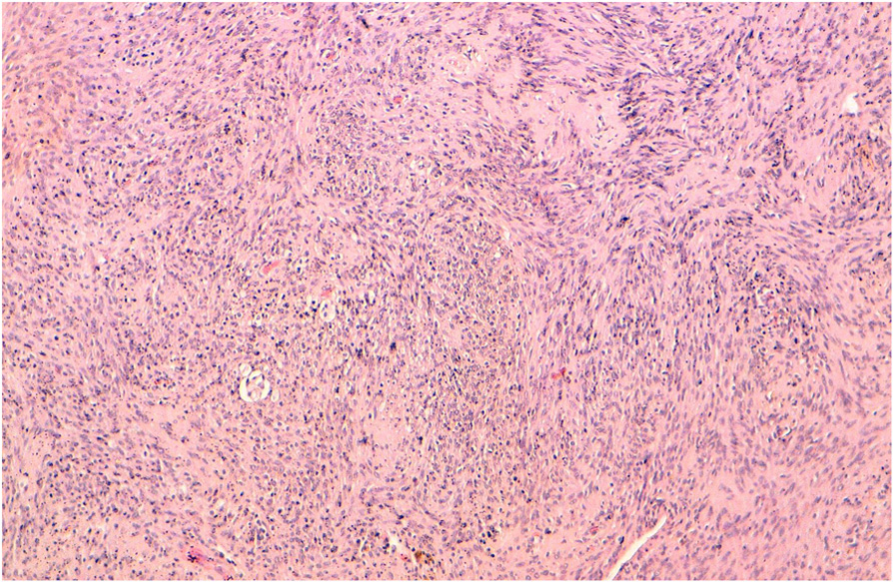
Fibrous capsule with the underlying tumor exhibiting cellular and hypocellular area, Nuclei of the spindle-shaped cells arranged in characteristic “Verocay bodies” (case 1).

**Figure 3:**
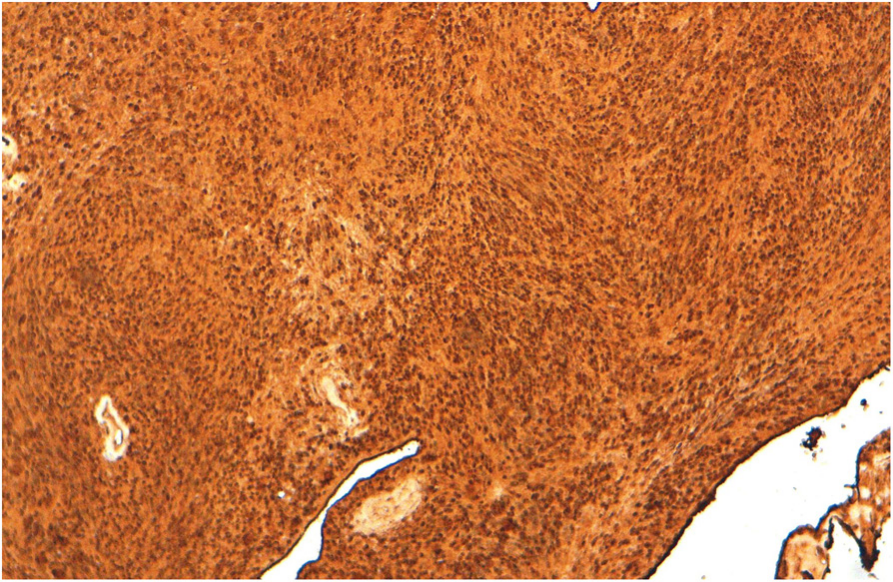
Cytoplasmic and nuclear immunostaining of neoplastic cells with protein S-100 (case 1).

Total conservative parotidectomy was done. The branches of the facial nerve were identified. Intra-operatively, a cystic mass with solid component was found involving the parotid gland. The surgical specimen of 2.8 × 1, 8 × 0, 8 cm and 4, 5 × 4, 5 × 2 cm with a cystic appearance was examined. Histopathology showed spindle-shaped cells arranged in characteristic Verocay bodies (Figure 2). The neoplastic cell showed nuclear and cytoplasmic immunopositivity with protein S-100 (Figure 3) and negative for smooth muscle actin and cytokeratin AE1/AE3. A diagnosis of schwannoma with cystic changes was confirmed.

### Case 2

**Figure 4:**
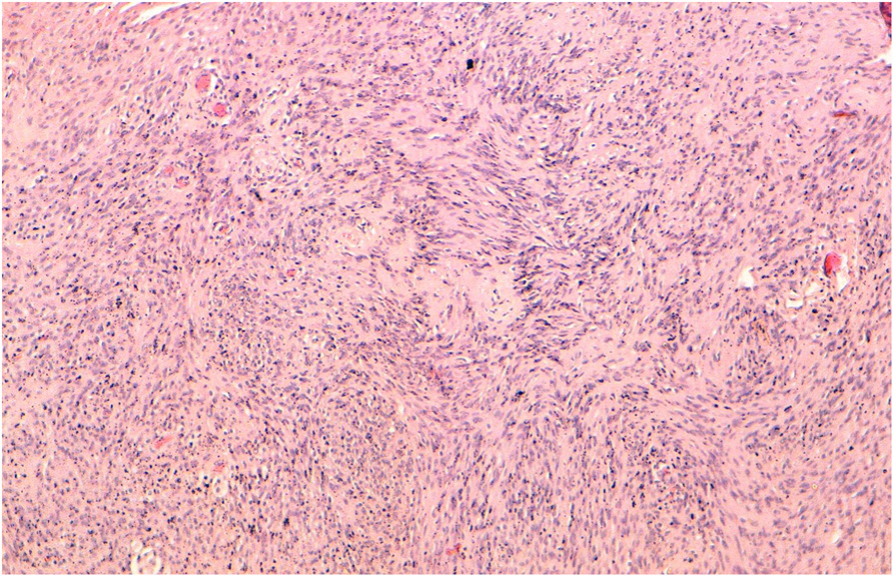
Benign proliferation of spindle-shaped cells arranged in characteristic “Verocay bodies” (Hematoxyline, Eosin, Safran × 100) (case 2).

**Figure 5:**
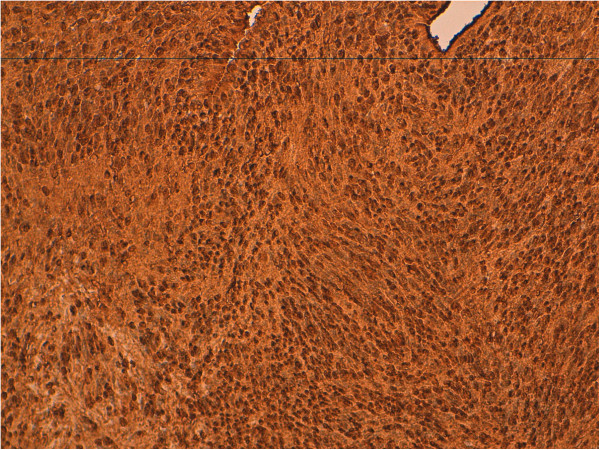
Cytoplasmic and nuclear immunostaining of neoplastic cells with protein S-100 (case 2).

A 75-year-old man with episodes of nasal obstruction, rhinorrhea, epistaxis, anosmia and headache. CT scan showed a large polypoid mass in the maxillary sinus and prolapsed into ipsilateral nasal cavity. Magnetic resonance imaging (MRI) scan revealed an enhancing, cystic mass in the right nasal cavity measuring 7 × 5 × 3 cms hypointense on signal intensity area on T1-weighted images, and high intensity area on T2-weighted images. The tumor expands into the orbit and nasopharynx. Hitological study of the specimen revealed a composed of cellular Antoni A areas with Verocay bodies and hypocellular myxoid Antoni B areas. The cells are fusiform with elongated fribillary cytoplasm, and buckled to spindled nuclei which show little pleomorphism, There are frequently small to medium-sized vessels with hyalinization in the Antoni B areas (Figure 4). The tumor cells are strongly and diffusely immunoreactive for S100 protein (Figure 5). They are negative for cytokeratine, muscular antibodies and CD34. Based on this data, the diagnosis of schwannoma was retained.

## Discussion

The head and neck regions accounts for 25 to 45 percent of benign schwannomas, and most of these arise in the eighth nerve but are relatively uncommon from the seventh or the fifth nerves. Neurogenic tumors found are mainly neurofibromas and schwannomas (Neurilemmomas). They represent a pathology, which is often not taken into account during clinical practice [3].

Schwannoma of the parotid gland can arise either from the facial nerve (7^th^) or from any other peripheral nerve running within the gland and it presents clinically as a primary salivary gland tumor [4]. Sinonasal schwannomas arise from the branches of the trigeminal (5^th^) nerve and autonomic nervous system, and most commonly involve the ethmoid and maxillary sinuses, followed by the nasal cavity, sphenoid and frontal sinuses. Cellular schwannoma tends to be located in the midline [2].

Schwannoma of head and neck regions seems to affect patients in their fifth decade. Mean ages of 42 years, 44 years and 45 years were reported by Liu [5], Caughey [6] and Marco [7] respectively. No characteristic symptom profile exists in intraparotid or nasal schwannoma. However, slow-growing, pain, tenderness, facial spasm or paralysis may be present in the first one [8]. The presenting symptoms of second one include obstruction, rhinorrhea, epistaxis, anosmia, headache, dysphagia, hearing loss, facial or orbital swelling, and pain.

The tumor typically presents either as a localized lesion or as part of a generalized syndrome of neurofibromatosis generally known as neurofibromatosis type-1 (NF1) or von Recklinghausen disease of the skin which is associated with somatic mutations at the NF1 gene, a tumor suppressor gene located in the pericentromeric region of chromosome 17 [9].

Grossly, schwannomas are solitary, encapsulated tumors usually attached to or surrounded by nerve. It appears to push axons aside and degenerative changes like cystic alterations or hemorrhagic necrosis are usually present [10].

The critical step in the management of neurogenic tumors of head and neck is the diagnosis. They may be also discovered incidentally on imaging studies.

The use of fine-needle aspiration (FNA) to aid in the diagnosis is a common and appropriate practice in parotid schwannoma [11,12]. The cytology may reveal spindle-shaped cells with ill-defined cytoplasm, arranged in clusters (Verocay bodies). In most cases, results are inconclusive [6].

Hence, a negative FNA finding alone should not delay or hinder surgical intervention when it is otherwise clinically indicated.

Magnetic resonance imaging (MRI) with gadolinium is the study of choice for imaging the suspected nerve lesions of the parotid and nasal cavity. On T1-weighted images, the main trunk of the nerve appears hypointense, with hyperintense surrounding fat tissue, it appears on high intensity within T2-weighted images [13].

Macroscopically, sinonasal and intraparotid schwannomas range in size up to 7 cm. they are a well-delineated but non-encapsulated globular, firm to rubbery yellow-tan mass. The cut surfaces show tan-grey, yellowish, solid to myxoid and cystic tissue, commonly with haemorrhage [2].

Histologically, two microscopic patterns of schwannomas exist, Antoni A and Antoni B. Antoni A lesions are characterized by broad interlacing ribbons of extended spindle cells with elongated nuclei arranged in waves, drifts and whorls. On cross section, these cylindrical cells produce a palisading pattern of nuclei about a central mass of cytoplasm called a Verocay body. Antoni B pattern is made up of very loose tissue, lacking the arrangement in the bundle and palisades, and is thought to be a degenerative form of Type A with a looser texture and polymorphism of cells separated by abundant myxoid, often microcystic matrix [10].

Mitoses are usually absent and malignant transformation of schwannoma is exceptionally rare [7]. Immunostaining for S-100 is required to establish the neural origin of the tumor, and smooth muscle actin (SMA) to rule out a leiomyoma such as our patients.

The differential diagnoses of intraparotid schwannoma should comprise the large diagnostic spectrum in which spindle cells are involved such as neurofibrome, fibroblastic/myofibroblastic tumors, most frequently nodular fasciitis, and fibromatosis with an infrequent myofibromatosis, fibroma, haemangiopericytoma, solitary fibrous tumor or inflammatory pseudotumor (inflammatory myofibroblastic tumor) [14].

Intranodal palisaded myofibroblastoma is another tumor that must be taken into account in differential diagnosis [15].

Like a cystic mass of the parotid, the most differential diagnoses include retention cysts, post-traumatic sialoceles, Warthin’s tumor, mucoepidermoid carcinoma and necrotic metastases [14]. The differential diagnoses of nasal schwanoma include other mesenchymal benign spindle cell tumor like fibroma, leiomyoma, nodular fasciitis and inflammatory pseudotumor. other pathological differential diagnoses of our case would include hybrid peripheral nerve sheath tumors, perineurioma, cellular neurothekeoma, nerve sheath myxoma (classic neurothekeoma), desmoplastic neurothekeoma, superficial angiomyxoma (cutaneous myxoma) [16], solitary neurofibroma with prominent differentiation of Meissner bodies, or spindle cell carcinoma [9,16].

Benign facial and trigeminal nerves schwannomas grow slowly and resection is not always indicated. Several authors have reported the occurrence of facial paralysis or several nasal symptoms when a simple biopsy or careful resection with apparent preservation of the function nerve is indicated [2,6].

## Conclusion

Schwannoma should be considered in the diagnosis of slowly growth lesions involving the parotid gland and nasal cavity.

## Consent

Written informed consent was obtained from each patient for publication of this report and any accompanying images.

## Abbreviations

FNA: Fine-needle aspiration; MRI: Magnetic resonance imaging; SMA: Smooth muscle actin.

## Competing interests

The authors declare that they have no competing interests.

## Authors’ contributions

IK, CL, HN and EH participated in the histopathological evaluation, performed the literature review, acquired photomicrographs and drafted the manuscript. HT and AA conceived and designed the study, gave the final histopathological diagnosis and revised the manuscript for important intellectual content. All authors read and approved the final manuscript.
